# On-demand PEGylation and dePEGylation of PLA-based nanocarriers *via* amphiphilic mPEG-*TK*-Ce6 for nanoenabled cancer chemotherapy

**DOI:** 10.7150/thno.37128

**Published:** 2019-10-22

**Authors:** Yueqiang Zhu, Chao Chen, Ziyang Cao, Song Shen, Laisheng Li, Dongdong Li, Junxia Wang, Xianzhu Yang

**Affiliations:** 1 School of Food and Biological Engineering, Hefei University of Technology, Hefei, Anhui, 230009, China; 2 Institutes for Life Sciences, School of Medicine, South China University of Technology, Guangzhou, Guangdong 510006, China; 3 Department of Laboratory Medicine, The First Affiliated Hospital of Sun Yat-sen University, Guangzhou, 510080, People's Republic of China

**Keywords:** PEGylation, dePEGylation, photocleavable molecule, nanocarrier, cancer therapy

## Abstract

**Rationale**: PEGylation of nanocarriers could extend blood circulation time and enhance tumor accumulation *via* the enhanced permeability and retention (EPR) effect. Unfortunately, the PEG moiety suppresses tumor cell internalization of nanocarriers, resulting in limited therapeutic efficiency (known as the PEG dilemma). Designing stimuli-responsive shell-detachable nanocarriers, which could detach the PEG corona from the nanocarriers in desired tumor tissues in response to the local environment, is an appealing approach to overcome the PEG dilemma, but nanocarrier applications are also limited by a lack of universal stimuli for PEG detachment.

**Methods**: In this study, we synthesized red light-responsive, amphiphilic mPEG bridged to the photosensitizer Ce6 *via* a thioketal (*TK*) bond (mPEG-*TK*-Ce6), which was then used to achieve the PEGylation of polylactide (PLA)-based nanoparticles encapsulating the Pt(IV) prodrug. The therapeutic efficacy of the prepared nanoparticles was evaluated *in vitro* and *in vivo*.

**Results**: We demonstrated that the amphiphilic mPEG-*TK*-Ce6 can realize the PEGylation of Pt(IV) prodrug-loaded PLA nanoparticles and consequently enhanced nanoparticle accumulation in tumor tissues. When the tumor tissues were subjected to 660 nm irradiation, reactive oxygen species (ROS) generated by Ce6 induced the rapid degradation of the adjacent *TK* bond, resulting in PEG detachment and enhanced tumor cell internalization. Therefore, mPEG-*TK*-Ce6 facilely achieved PEGylation and light-responsive dePEGylation of the nanocarrier for enhanced antitumor efficacy in nanomedicine.

**Conclusion**: Such red light-responsive amphiphilic mPEG-*TK*-Ce6 facilely achieved PEGylation and dePEGylation of the nanocarrier, providing a facile strategy to overcome PEG dilemma.

## Introduction

Coating the surface of antitumor nanocarriers with polyethylene glycol (PEG), i.e., PEGylation, is the most widely used strategy to inhibit protein adsorption and prevent rapid elimination 1-5, consequently extending the blood circulation time, enhancing tumor accumulation *via* the enhanced permeability and retention (EPR) effect, and decreasing collateral damage to normal healthy tissues. Several PEGylated nanomedicines, including PEGylated liposomal doxorubicin (Doxil^®^) and the PEG-PLA micelle form of paclitaxel (Genexol-PM^®^), have been approved for the treatment of malignant tumors 6-9, and many PEGylated nanodrugs have entered clinical trials. As expected, PEGylation has achieved improved tumor enrichment of nanoparticles after systemic administration; however, PEGylation also hinders the internalization of nanocarriers, resulting in limited therapeutic efficiency 10-13. Therefore, it is highly desirable to develop a facile strategy to achieve the on-demand PEGylation and dePEGylation of nanocarriers according to the requirements.

An appealing approach for overcoming the PEG dilemma is the design of shell-detachable nanocarriers, which detach the PEG corona from nanocarriers in the tumor tissues in response to the local environment, subsequently promoting cellular uptake and enhancing antitumor efficacy 14-18. Therefore, endogenous stimuli in the tumor microenvironment, such as acidic pH 19-21, hypoxia 22-23, and overexpression of certain enzymes 24-25, have been utilized to develop PEG-detachable nanoparticles. However, due to the heterogeneity of tumors, the pH of the extracellular environment and the dynamically varied expression of enzymes in different types of tumors in different stages or in different parts of the same tumor 26-28, the broader applicability of tumor microenvironment-responsive nanoparticles is limited. Thus, exploring alternative and generally applicable strategies for tumor-specific PEG detachment is urgently needed.

**Figure 1:**
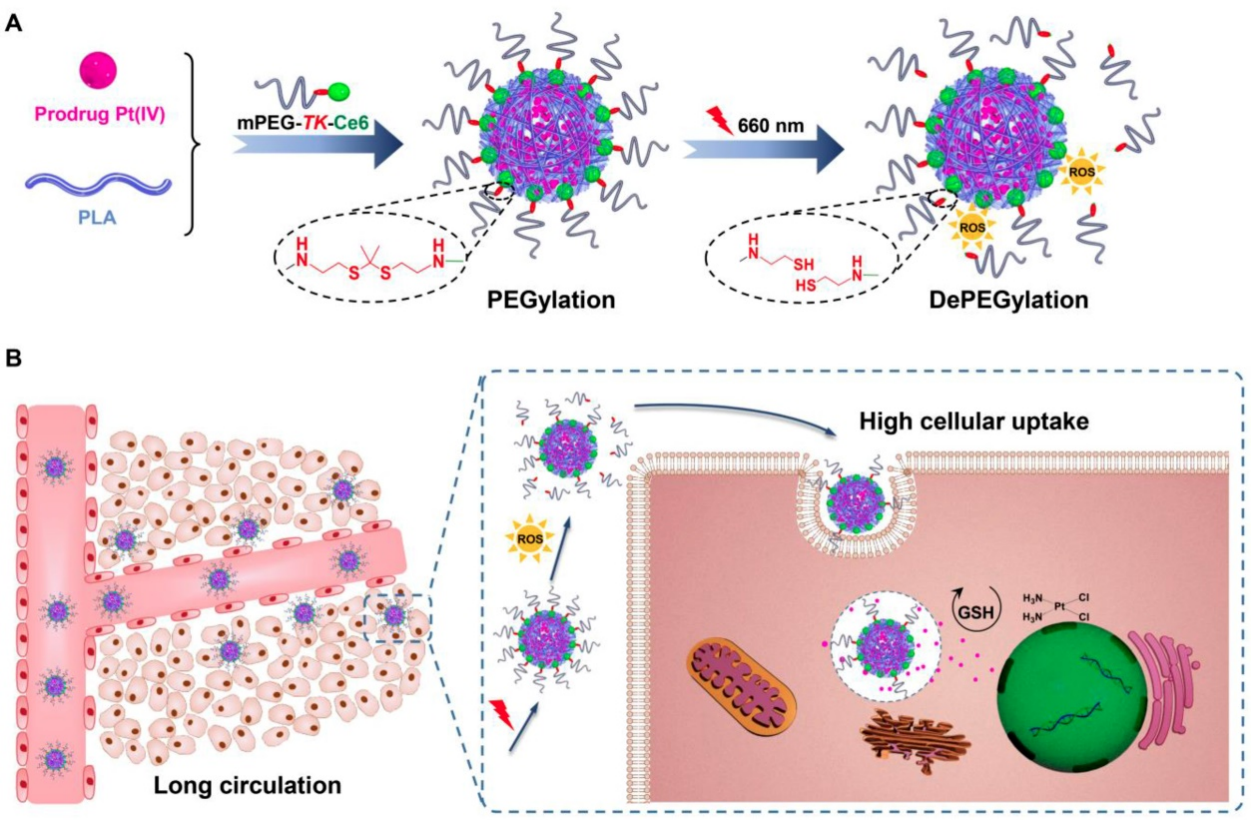
(A) Schematics showing the on-demand PEGylation and dePEGylation of nanocarriers *via* amphiphilic mPEG-*TK*-Ce6. (B) The PEGylation of TCNP_Pt_ efficiently prolonged the circulation time. Then, the dePEGylation of TCNP_Pt_ at the tumor site was achieved by cleaving the adjacent *TK* bond of mPEG-*TK*-Ce6 after irradiation to enhance cellular uptake.

In recent years, red and near-infrared (NIR) light (650-950 nm), which are triggers for photothermal and photodynamic therapy, have received tremendous attention because of several advantages 29-33. Light in the red and NIR regions is also considered an external stimulus and used in stimuli-responsive drug delivery systems 34-38. Recently, it has been reported that reactive oxygen species (ROS) generated by photosensitizers under irradiation readily cleave thioketal (*TK*) bonds 39-42. Therefore, an innovative molecule, amphiphilic mPEG bridged to the photosensitizer Ce6 *via* a *TK* bond (mPEG-*TK*-Ce6), was synthesized and used to achieve the PEGylation of polylactide (PLA)-based nanoparticles encapsulating the prodrug Pt(IV). The prepared nanocarrier is referred to as TCNP_Pt_. PEGylation protects nanocarriers from rapid elimination, consequently enhancing the extravasation of nanoparticles from vessels into tumor tissues. When the tumor was subjected to 660 nm irradiation, ROS generated by Ce6 induced the rapid degradation of adjacent* TK* bonds and dePEGylation of TCNP_Pt_, facilitating tumor cell internalization and improving chemotherapeutic effects (Figure 1). We systematically evaluated the effect of the on-demand PEGylation and dePEGylation of TCNP_Pt_ with the assistance of red light on the cellular uptake, blood circulation, tumor accumulation, and antitumor efficacy.

## Materials and Methods

### Preparation and characterization of TCNP_Pt_

To prepare Pt(IV) prodrug-encapsulated nanoparticles (TCNP_Pt_), mPEG_45_-*TK*-Ce6 (5.0 mg), PLA_40_ (5.0 mg, the synthesis described in supporting information) and Pt(IV) (2.5 mg) were dissolved in dimethyl sulfoxide (DMSO, 1.0 mL), and then added dropwise to ultrapure water (10.0 mL) for 2 h. This solution was dialyzed against ultrapure water for 24 h and filtered through a 0.45 μm filter. Similarly, fluorescein isothiocyanate (FITC)-labeled nanocarriers ^FITC^TCNP_Pt_ were fabricated by the addition of FITC-labeled PLA (0.2 mg) to the preparation. The size and morphology of TCNP_Pt_ were determined by light scattering (Malvern Zetasizer Nano ZS90) and transmission electron microscopy (TEM, JEOL-2010, Tokyo, Japan), respectively.

### Degradation of TCNP_Pt_ under 660 nm light

Aqueous TCNP_Pt_ (5.0 mL, [Ce6] = 150 μg/mL) solution was irradiated (660 nm, 0.05 W/cm^2^) for different amounts of time (0, 5, 10, and 20 min). Then, the samples were lyophilized for gel permeation chromatography (GPC) and ^1^H NMR analysis. The degradation ratios of the *TK* bond were calculated according to previous reports 43.

### *In vitro* cellular uptake of ^FITC^TCNP_Pt_ with preirradiation

After receiving preirradiation (660 nm and 0.05 W/cm^2^ for 5, 10 or 20 min), the ^FITC^TCNP_Pt_ nanocarriers (0.1 mL, [FITC-PLA]= 2 μg/mL, [platinum]= 4.31 μg/mL) were cocultured with MDA-MB-231 cells. The cells were analyzed by flow cytometry (BD Biosciences, Franklin Lakes, NJ) at 2 and 4 h. In addition, portions of the samples were lyophilized for inductively coupled plasma mass spectrometry (ICP-MS) analysis of the intracellular platinum concentration. For confocal laser scanning microscopy (CLSM) observations, the treated cells were counterstained with Alexa Fluor568 phalloidin and 4′,6-diamidino-2-phenylindole (DAPI) at 4 h and then analyzed by CLSM (LSM 710, Carl Zeiss, Inc., Jena, Germany).

### *In vitro* antiproliferation assay

The TCNP_Pt_ nanocarriers were preirradiated as described above and cocultured with MDA-MB-231 cells at different platinum concentrations (0.5, 1.0, 2.0 and 4.0 μM). After further incubation for 24 h, a 3-(4,5-dimethylthiazol-2-yl)-2,5-diphenyltetrazolium bromide (MTT) assay was performed to analyze cell viability. After the aforementioned treatment was administered at a platinum concentration of 2.0 μM, cell apoptosis was also examined by flow cytometric analysis.

### *In vivo* and* ex vivo* distribution of TCNP_Pt_

Mice bearing MDA-MB-231 xenografts were treated with TCNP_Pt_ *via* intravenous (*i.v.*) injection and then imaged at predetermined time points by the Bruker Xtreme In-Vivo Fluorescence Imaging System (excitation, 650 nm). The injection dosages of platinum and Ce6 were 2.0 and 3.48 mg/kg, respectively. Moreover, after *i.v.* injection of TCNP_Pt_ for 6 h, the tumor site of the TCNP_Pt_ (L+) group was irradiated (660 nm and 0.05 W/cm^2^ for 20 min). The main organs and tumors were collected at 24 h for fluorescent imaging and ICP-MS analyses.

### Therapeutic efficacy *in vivo*

Mice bearing MDA-MB-231 xenografts were randomly divided into the following 6 groups (five mice per group): PBS, cisplatin, Pt(IV) prodrug-free TCNP plus irradiation (TCNP (L+)), TCNP_Pt_ without irradiation (TCNP_Pt_ (L-)), TCNP_Pt_ plus irradiation (TCNP_Pt_ (L+)) and TCNP (L+) + TCNP_Pt_. The equivalent injection doses of platinum and Ce6 were 2.0 and 3.48 mg/kg, respectively. The tumor sites in the predetermined groups were irradiated (660 nm and 0.05 W/cm^2^ for 20 min) at 6 h postinjection. For the TCNP (L+) + TCNP_Pt_ group, TCNPs were injected (*i.v.*), the tumor was irradiated at 6 h postadministration, and TCNP_Pt_ was then injected. On the 16th day following the first treatment, the tumor tissues were excised, weighed and imaged.

## Results and discussion

### Characterization of red light-responsive TCNP_Pt_

To synthesize amphiphilic mPEG-*TK*-Ce6, carboxyl-terminated mPEG_45_-COOH was first reacted with a *TK* linker and subsequently conjugated with Ce6 (Figure S1). A PLA homopolymer was synthesized using 10-hydroxydecanoic acid as the initiator under catalysis by Sn(Oct)_2_ (Figure S2A). Then, the amphiphilic mPEG-*TK*-Ce6 with a critical aggregation concentration of 0.036 mg/mL (Figure S3) was utilized to achieve the PEGylation of the Pt(IV) prodrug-loaded PLA nanoparticle (denoted TCNP_Pt_). The loading contents of Ce6 and platinum in TCNP_Pt_ were 7.5 ± 0.59% and 4.31 ± 0.36%, respectively. Similarly, FITC-labeled TCNP_Pt_ (^FITC^TCNP_Pt_) was prepared to track the nanocarrier (Figure S2C).

**Figure 2:**
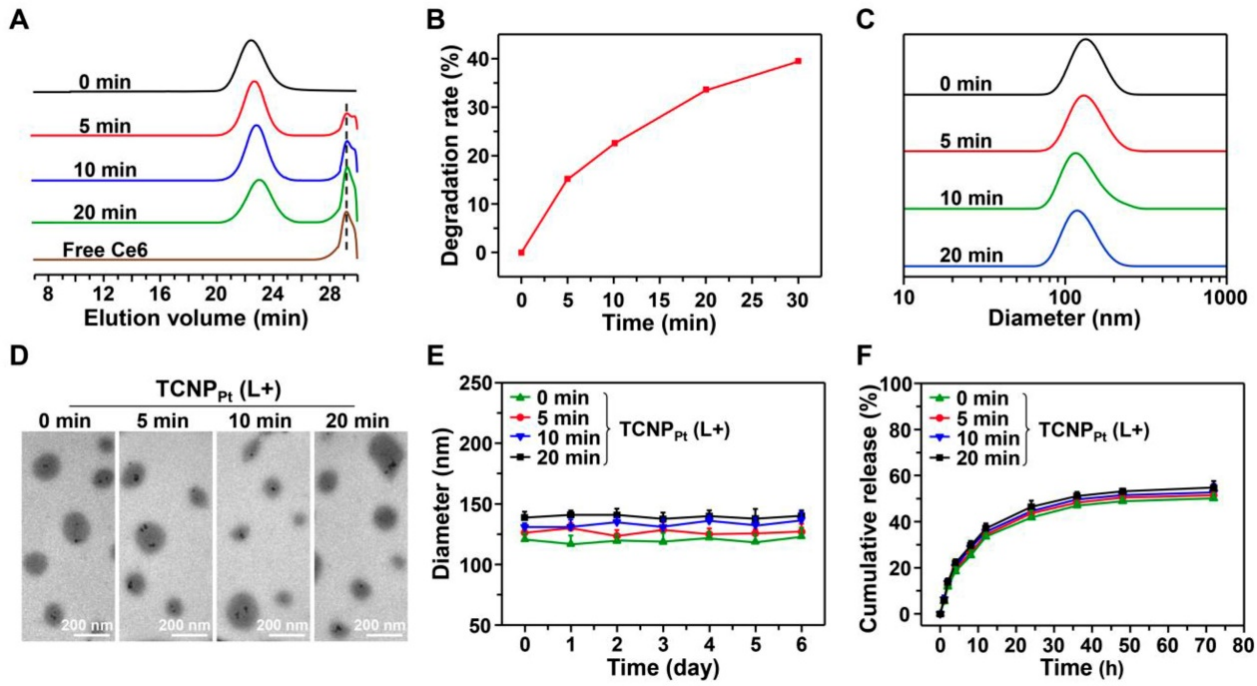
** Characterization of red light-responsive TCNP_Pt_.** (A) GPC of mPEG-*TK*-Ce6 after irradiation with a 660 nm laser for different amounts of time (0.05 W/cm^2^). (B) The degradation of mPEG-TK-Ce6 with respect to irradiation for different amounts of time. The size (C), TEM images (D), and stability (E) of TCNP_Pt_ after irradiation for different amounts of time. (F) The platinum release curve of TCNP_Pt_ after 660 nm light irradiation for different amounts of time.

According to our design, 660 nm irradiation induced the production of ROS by the conjugated Ce6 (Figure S4), causing the cleavage of adjacent* TK* bonds and rapid dePEGylation. To demonstrate this, TCNP_Pt_ was irradiated by light (660 nm and 0.05 W/cm^2^ for 5, 10 or 20 min), and the TCNP_Pt_ nanoparticles were then collected for GPC analysis. As shown in Figure 2A, the peak gradually increased at 29.2 min (Ce6) as the irradiation time increased. In addition, the TCNP_Pt_ nanoparticles were lyophilized and analyzed by ^1^H NMR after irradiation. As the preirradiation time increased, the amount of CH_3_ from the *TK* bond of mPEG-TK-Ce6 (with a peak at 1.58 ppm) gradually decreased (Figure S5), while the peak at 2.12 ppm indicated that the amount CH_3_ from acetone (the product of *TK* bond rupture) increased. Based on these ^1^H NMR spectra, the degradation ratio of the *TK* bond was calculated, as shown in Figure 2B, and found to exhibit time dependence. Approximately 39.6% of the *TK* bond of mPEG-*TK*-Ce6 was cleaved after receiving 0.05 W/cm^2^ irradiation for 30 min. These results indicated that a 660 nm laser efficiently caused PEG detachment under light irradiation.

The size, morphology, zeta potential and stability of TCNP_Pt_ with or without 660 nm light irradiation were also determined. TCNP_Pt_ nanoparticles were irradiated for different amounts of time (0, 5, 10, and 20 min at 0.05 W/cm^2^), and the nanoparticles that were not irradiated were used as a control. PEG detachment did not significantly affect the size, morphology and stability of TCNP_Pt_. The TCNP_Pt_ nanoparticles were approximately 120 nm in diameter (Figure 2C) and had a spherical morphology (Figure 2D). The size (Figure 2C) and zeta potentials (Figure S6) of TCNP_Pt_ exhibited slightly decrease after light irradiation. And, preirradiation also caused a slight increase in the TCNP_Pt_ size in 10% FBS within 6 days (Figure 2E), which could be attributed to the enhanced protein adsorption after dePEGylation. The dilution stability of TCNP_Pt_ were performed with the different dilution in PBS, which showed that TCNP_Pt_ maintain a stable form (Figure S7). In addition, compared with the nonirradiated condition, light-induced PEG detachment did not obviously affect the release rate of the Pt(IV) prodrug from TCNP_Pt_ (Figure 2F).

### Red light-induced dePEGylation enhances the cellular uptake of TCNP_Pt_

**Figure 3:**
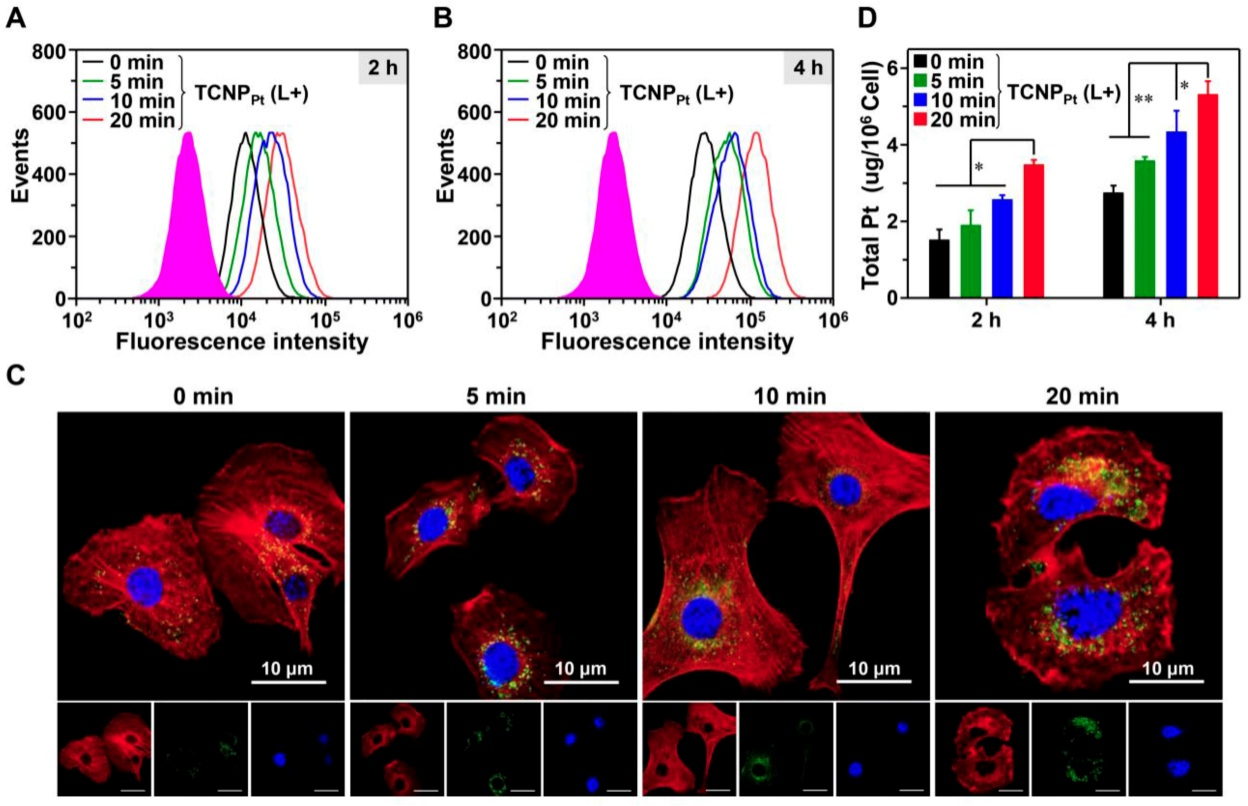
** Red light-induced dePEGylation enhances the cellular uptake of TCNP_Pt_.** The cellular uptake of preirradiated ^FITC^TCNP_Pt_ (0, 5, 10, and 20 min) analyzed using flow cytometry after 2 h (A) and 4 h (B) of incubation. CLSM images (C) and ICP-MS analysis (D) of cells after receiving these treatments. The green fluorescence represented the ^FITC^TCNP_Pt_ singal. Cell nuclei and the cytoskeleton were stained with DAPI (Blue) and Alexa Fluor568 phalloidin (Red), respectively. The scale bar is 10 μm.

The PEGylation of nanocarriers minimizes their elimination *in vivo*, and the disadvantage of PEGylation is the hindrance to cellular uptake. We proposed that red light-induced dePEGylation could overcome these limitations. To investigate this hypothesis, the cellular uptake of preirradiated ^FITC^TCNP_Pt_ was examined using qualitative and quantitative methods. First, ^FITC^TCNP_Pt_ was irradiated and then cocultured with MDA-MB-231 cells for 2 and 4 h (the dose of platinum was 4.31 μg/mL). Then, intracellular FITC fluorescence was analyzed by flow cytometry. As shown in Figure 3A and 3B, preirradiation of ^FITC^TCNP_Pt_ obviously elevated the intracellular fluorescence signal at 2 and 4 h, and showed that preirradiation had a time-dependent effect on cellular uptake. The enhanced tumor cell uptake of the preirradiated nanoparticles was further corroborated by CLSM. Clearly, the most intracellular green fluorescence was observed when the cells were treated with ^FITC^TCNP_Pt_ plus 20 min of preirradiation (Figure 3C). Furthermore, we also determined the intracellular platinum content *via* ICP-MS. At both timepoints, preirradiation significantly elevated the intracellular platinum concentration (Figure 3D). For instance, the amount of intracellular platinum in cells treated with ^FITC^TCNP_Pt_ plus 20 min of preirradiation was 1.96-fold greater than that in cells treated with ^FITC^TCNP_Pt_ without preirradiation after 4 h of incubation. Thus, these results demonstrated that light-induced dePEGylation led to enhanced cellular uptake of TCNP_Pt_.

### Red light-induced dePEGylation significantly enhances the antiproliferative activity

**Figure 4:**
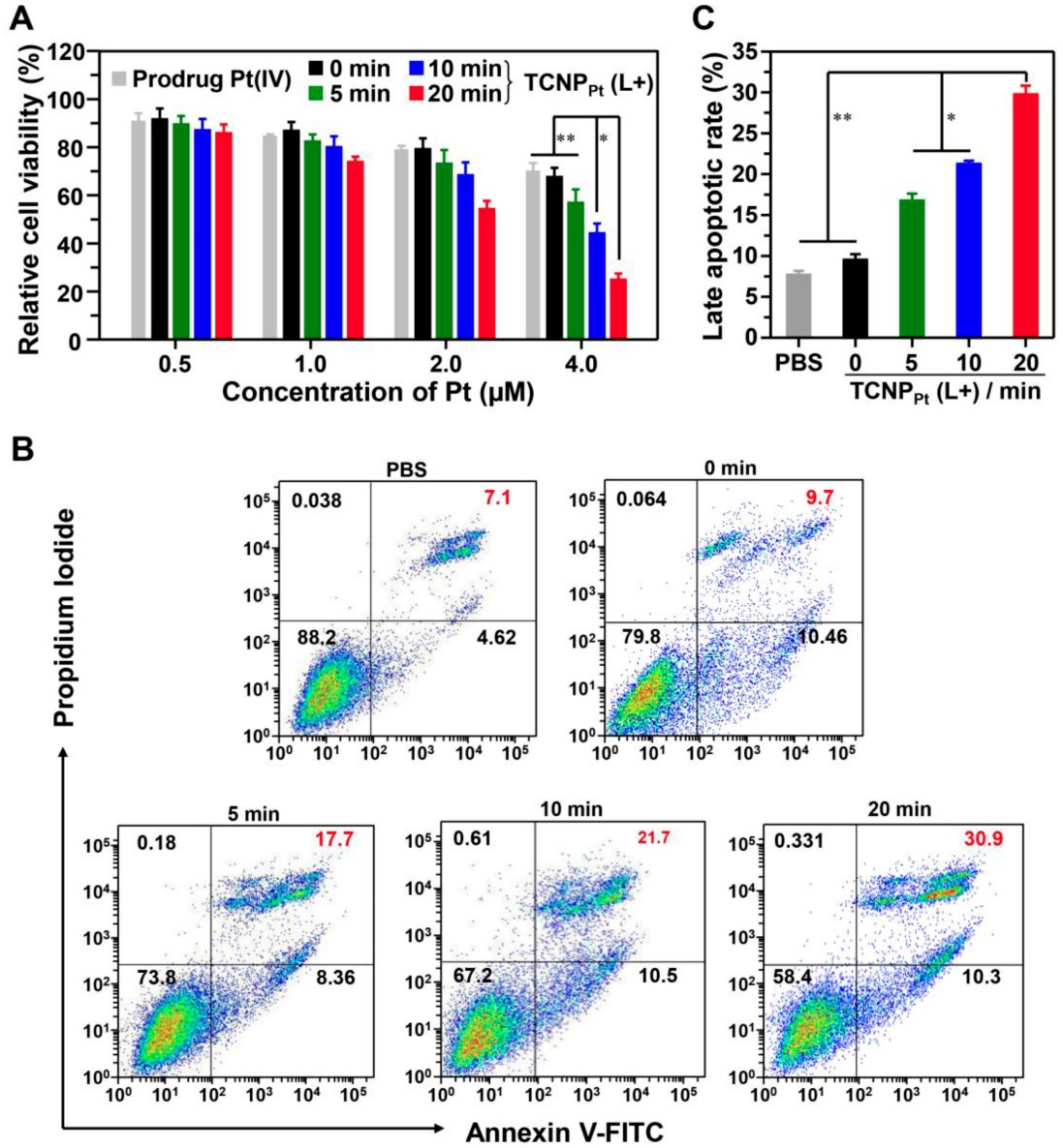
** Red light-induced dePEGylation significantly enhances the antiproliferative activity.** (A) Cell viability of preirradiated TCNP_Pt_ (0, 5, 10, and 20 min) at different concentrations of TCNP_Pt_. (B) Cell apoptosis induced by preirradiated TCNP_Pt_ (0, 5, 10, and 20 min) at an identical platinum concentration of 2.0 μM. (C) The percentage of late apoptotic cells induced by different treatments according to flow cytometric analysis (Figure 4C).

We demonstrated that light-induced dePEGylation increased the amount of intracellular platinum. To further investigate the advantage of red light-responsive nanocarriers in cancer therapy, their antiproliferative activity against tumor cells was examined. TCNP_Pt_ was preirradiated for different periods, and then various amounts of TCNP_Pt_ were used to treat the tumor cells for the MTT assay. Figure 4A showed that tumor cell proliferation was most significantly inhibited by TCNP_Pt_ plus 20 min of preirradiation at platinum concentrations of 2.0 μM or 4.0 μM. Noted that the blank nanoparticle TCNP did not exhibit obvious toxicity (Figure S8). Furthermore, after receiving these treatments, the cells were stained with annexin V-FITC and propidium iodide (PI) for the analysis of cell apoptosis. At the platinum concentrations shown in Figure 4B and 4C, TCNP_Pt_ without irradiation had a negligible effect on the survival of tumor cells; however, preirradiation for 5 and 10 min induced 17.7% and 21.7% cell apoptosis, respectively. Preirradiation of TCNP_Pt_ for 20 min significantly increased the percentage of apoptotic cells (30.9%). Collectively, these anticancer results verified that the red light-induced dePEGylation effect markedly increased the antiproliferative activity of the tumor cells.

### Red light-induced dePEGylation improved tumor accumulation

**Figure 5:**
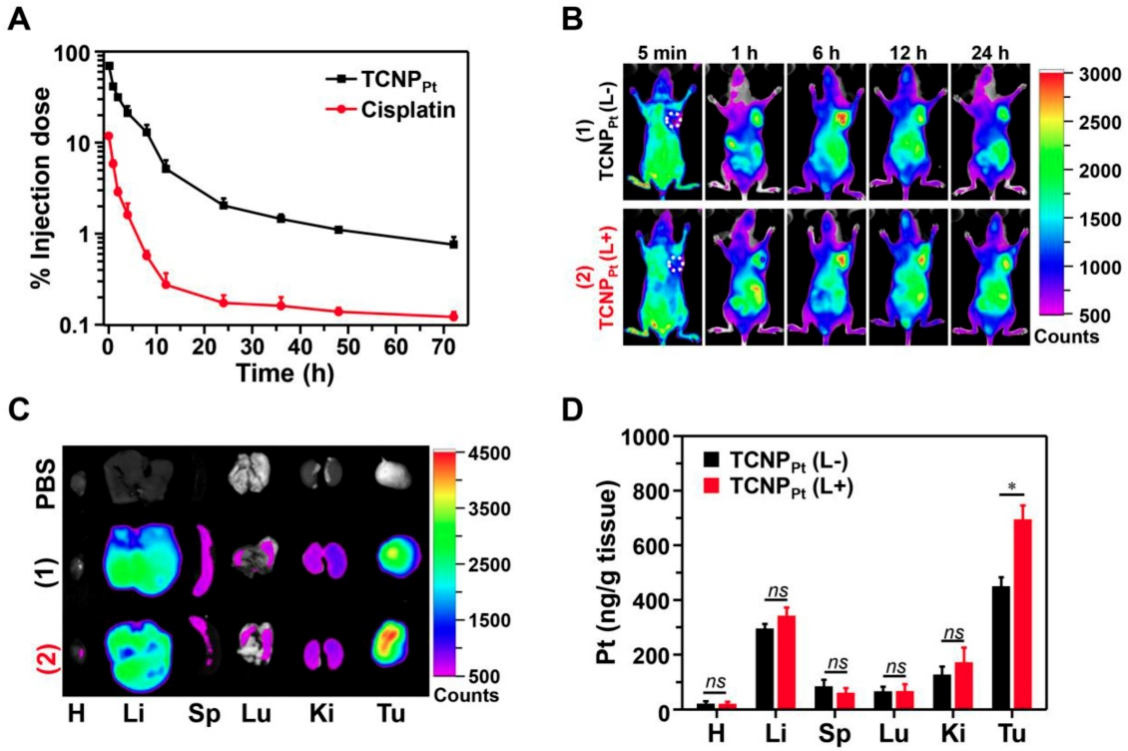
** Red light-induced dePEGylation improved tumor accumulation**. (A) The platinum concentration over time for free cisplatin and TCNP_Pt_. (B) The distribution of TCNP_Pt_ in MDA-MB-231 tumor-bearing mice. (C) *Ex vivo* images of the main organs at 24 h. (D) ICP-MS analysis of the platinum content in the major organs. In Figure 5B-D, TCNP_Pt_ (L+) indicates that the tumor site was irradiated at 6 h.

Encouraged by the superior *in vitro* antiproliferation efficacy, animal experiments were performed to investigate the potential advantage of PEGylation during blood circulation and subsequent dePEGylation at the tumor site of TCNP_Pt_ with the assistance of light. Before evaluating the antitumor efficacy, the pharmacokinetics and biodistribution of TCNP_Pt_ were examined. ICR mice were injected (*i.v.*) with TCNP_Pt_ and cisplatin at an identical platinum dose of 2.0 mg/kg, and the concentration of platinum in plasma over time was analyzed (Figure 5A), and the pharmacokinetic parameters were calculated in Table S1. Clearly, free cisplatin was rapidly eliminated in the blood circulation, while the circulation of TCNP_Pt_ was significantly prolonged due to PEGylation.

Because TCNP_Pt_ with prolonged blood circulation was readily extravasated from vessels into the tumor tissues *via* the EPR effect, the biodistribution of TCNP_Pt_ was evaluated in the mice bearing MDA-MB-231 xenografts. As shown in Figure 5B, an obvious fluorescence signal was observed at the tumor sites at 1 h postinjection, and the intensity of the signal was strong until 24 h postinjection. To further verify whether the dePEGylation of TCNP_Pt_ induced by red light enhances TCNP_Pt_ tumor cell uptake and improves the subsequent accumulation in the tumor tissue, tumor-bearing mice were administered TCNP_Pt_, and portions of the mice tumor tissues were irradiated at 6 h postinjection. The main organs were collected and observed at 24 h postinjection. As shown in Figure 5C and Figure S9, the fluorescence signal observed in the tumors in the TCNP_Pt_ (L+) group was greater than that in the TCNP_Pt_(L-) group, but the signal intensity of the organs in both groups was similar, indicating that the dePEGylation induced by red light enhances the internalization of the nanoparticles by the tumor cells. In addition, the platinum content at 24 h after administration was examined using ICP-MS, as shown in Figure 5D. The platinum content in the tumor tissue in the TCNP_Pt_ (L+) group was 1.64-fold greater than that in the TCNP_Pt_ (L-) group. Therefore, the on-demand PEGylation and dePEGylation of TCNP_Pt_ assisted by light not only enhances drug accumulation in tumor tissues but also potentially reduces nonspecific cellular uptake and avoids cytotoxicity.

### The on-demand PEGylation and dePEGylation of TCNP_Pt_ assisted by light significantly improves the anticancer efficacy

**Figure 6:**
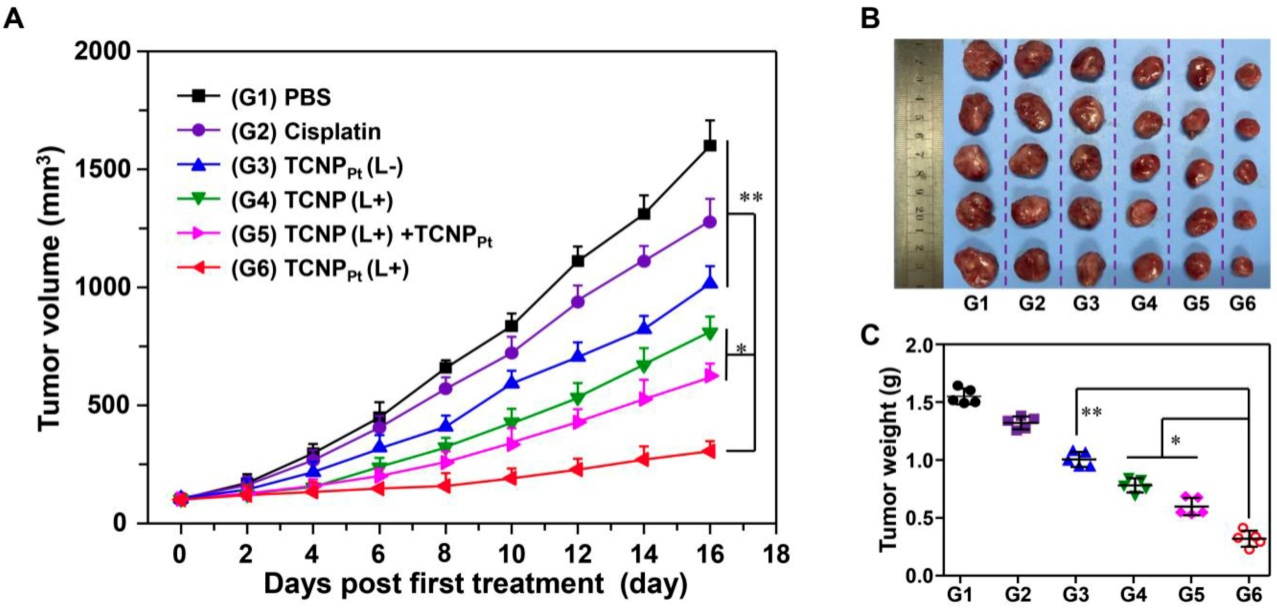
** The on-demand PEGylation and dePEGylation of TCNP_Pt_ assisted by light significantly improves the anticancer efficacy.** (A) Tumor growth curves after receiving different treatments. The mice were divided into the following six groups: PBS, cisplatin, Pt(IV) prodrug-free TCNP plus irradiation (TCNP (L+)), TCNP_Pt_ without irradiation (TCNP_Pt_ (L-)), TCNP_Pt_ plus irradiation (TCNP_Pt_ (L+)) and TCNP (L+) plus TCNP_Pt_ (L-). Tumor images (B) and weights (C) of the various groups on the 16^th^ day.

The abovementioned results clearly showed the advantage of on-demand PEGylation and dePEGylation of TCNP_Pt_ assisted by light. It is rational to speculate that tumor site-specific light irradiation significantly increases the anticancer efficacy of TCNP_Pt_. Therefore, mice bearing MDA-MB-231 xenografts were randomly divided into the following 6 groups (n = 5): PBS, cisplatin, platinum-free TCNP plus irradiation (TCNP (L+)), TCNP_Pt_ without irradiation (TCNP_Pt_ (L-)), TCNP_Pt_ plus irradiation (TCNP_Pt_ (L+)) and TCNP (L+) plus TCNP_Pt_ (L-). The mice that were irradiated received the irradiation treatment at 6 h postinjection. As shown in Figure 6A, the cisplatin and TCNP_Pt_ (L-) treatments only slightly inhibited tumor growth. In addition, the TCNP (L+) treatment exhibited moderate anticancer efficacy, which could be due to the photodynamic effect of the conjugated Ce6. Furthermore, the tumor-suppressive effect of TCNP_Pt_ with light irradiation was much better than the combination of TCNP (L+) and TCNP_Pt_ (L-), verifying that red light-induced PEG detachment significantly enhances the anticancer efficacy of TCNP_Pt_. Notably, no obvious body weight loss was observed (Figure S10). In addition, the tumor images (Figure 6B) and tumor weights (Figure 6C) showed that the TCNP_Pt_ (L+) group had the best anticancer efficiency.

## Conclusion

We successfully synthesized red light-responsive, amphiphilic mPEG-*TK*-Ce6 for the on-demand PEGylation and dePEGylation of the TCNP_Pt_ nanocarrier. The PEGylation of TCNP_Pt_ efficiently prolonged its blood circulation after systemic administration. When a 660 nm laser was used to irradiate the tumor site, ROS generated by Ce6 in mPEG-*TK*-Ce6 cleaved the adjacent* TK* bond to cause the dePEGylation of TCNP_Pt_, significantly enhancing tumor cellular uptake and the subsequent anticancer effect. Importantly, compared with other stimuli-responsive nanocarriers, red light-responsive TCNP_Pt_ is more extensive and practical, and this study provides a facile strategy to achieve the on-demand PEGylation and dePEGylation of nanocarriers.

## Supplementary Material

Supplementary materials and methods, figures, and table.
