# Supplementary material for: On-demand PEGylation and dePEGylation of PLA-based nanocarriers via amphiphilic mPEG-TK-Ce6 for nanoenabled cancer chemotherapy
Source: Theranostics. 2019 Oct 22;9(26):8312–20. doi: 10.7150/thno.37128 (PMC6857060; doi:10.7150/thno.37128)
Supplement: Supplementary file 1 — Supplementary materials and methods, figures, and table. [file thnov09p8312s1.pdf]

Supporting Information

On-demand PEGylation and dePEGylation of PLA-based nanocarriers *via* amphiphilic mPEG-*TK*-Ce6 for nanoenabled cancer chemotherapy

Yueqiang Zhu[1,*], Chao Chen[1,*], Ziyang Cao[2,*], Song Shen[2,✉], Laisheng Li[3], Dongdong Li[2], Junxia Wang[2], Xianzhu Yang[2,✉]

1. School of Food and Biological Engineering, Hefei University of Technology, Hefei, Anhui, 230009, China

2. Institutes for Life Sciences, School of Medicine, South China University of Technology, Guangzhou, Guangdong 510006, China

3. Department of Laboratory Medicine, The First Affiliated Hospital of Sun Yat-sen University, Guangzhou, 510080, People's Republic of China

[*] These authors contributed equally to this work.

✉**Corresponding Authors:** E-mail: yangxz@scut.edu.cn (Xianzhu Yang), E-mail: shensong@scut.edu.cn (Song Shen)

Materials.

Cisplatin was purchased from Shandong Boyuan Pharmaceutical Co., Ltd. (Shandong, China). 3-(4, 5-Dimethylthiazol-2-yl)-2,5-diphenyl tetrazolium bromide (MTT) and 4,6-diamidino-2-phenylindole (DAPI) were purchased from Sigma-Aldrich (St. Louis, USA). Chlorin e6 (Ce6) was purchased from J&K Chemical Ltd. (Shanghai, China). Dichlorofluorescin diacetate (DCFH-DA) was purchased from Beyotime Biotechnology. Dulbecco's-modified eagle medium (DMEM) and fetal bovine serum (FBS) were purchased from Gibco BRL (Eggenstein, Germany). Cysteamine hydrochloride, methoxy and carboxyl terminated poly(ethylene glycol) (PEG) (Mn=2,000 g/mol) were purchased from Aladdin Chemical Co., Ltd. (Shanghai, China). *D*,*L*-lactide was purchased from Jinan Daigang Biomaterial Co. Ltd. (Jinan, China). The Pt(IV) prodrug *c,t,c*-$[Pt(NH_3)_2(O_2CCH_2CH_2CH_2CH_2CH_2CH_2CH_2CH_2CH_3)_2Cl_2]$ was synthesized according to a method reported in the literature.[1] Other organic solvents and reagents were used as received.

Synthesis of PEG_{45}-*TK*-NH_2.

2’-(propane-2, 2-diylbis (sulfanediyl)) bis (ethan-1-amine) were synthesized according to previous method.[2] Carboxyl terminated PEG ($mPEG_{45}$-COOH, 1.0 mmol, 2.0 g), dicyclohexylcarbodiimide (DCC, 1.1 mmol, 0.23 g), and *N*-hydroxysuccinimide (NHS, 1.1 mmol, 0.13 g) were dissolved in 10 mL CH_2Cl_2. After reacting for 6 h, the solution of NHS-activated PEG was dropwise added into 10 mL CH_2Cl_2 containing PDSE (3.0 mmol, 0.58 g) under stirring, and further reacted

for 24 h at room temperature (R.T.). And then, the mixture was filtered, concentrated and precipitated into a cold mixture of diethyl ether/methanol (10/1, v/v) twice to gain the crude ROS-sensitive macroinitiator mPEG$_{45}$-*TK*-NH_2. Then, the crude products were purified by a column chromatography (1.26 g, 58%).

Synthesis of mPEG$_{45}$-*TK*-Ce6.

Ce6 (0.54 mmol, 0.32 g), dicyclohexylcarbodiimide (DCC, 1.78 mmol, 0.37 g), and *N*-hydroxysuccinimide (NHS, 1.78 mmol, 0.21 g) were dissolved in 20 mL CH_2Cl_2. After reacting for 6 h, the solution of Ce6 was dropwise added into 20 mL CH_2Cl_2 containing mPEG-*TK*-NH_2 (0.49 mmol, 1.1 g) under stirring, and further reacted for 24 h at R.T. And then, the mixture was filtered, concentrated and precipitated into a cold mixture of diethyl ether/methanol (10/1, v/v) twice to gain the ROS-sensitive mPEG$_{45}$-*TK*-Ce6 (1.09 g, 78.6%).

Synthesis of PLA homopolymer and FITC-PLA.

PLA polymer was synthesized using 10-hydroxydecanoic acid as the initiator under the catalysis $Sn(Oct)_2$. In a typical procedure, anhydrous 10-hydroxydecanoic acid (0.4 mmol, 0.075 g), L-lactide (13.9 mmol, 2.0 g) were dissolved in anhydrous methylbenzene (15 mL) in a fresh flamed flash. Stirring for 30 min at 80 °C until the solid is completely dissolved, then $Sn(Oct)_2$ (0.04 mmol, 16 mg) was added to the mixture. The reaction was carried on at 80 °C for 3 h. Then benzoic acid (0.6 mmol, 73 mg,) was added to terminate the reaction, and the mixture was concentrated and precipitated into a cold diethyl ether/methanol mixture (10/1, v/v) twice. The product was dried under vacuum at to obtain the product with a yield of 69.1% (1.38 g), and

the degrees of polymerization of the obtained PLA was 40 according to its ^{1}H NMR, denoted as PLA_{40}. Then, the FITC labeled PLA homopolymer (FITC-PLA) was obtained accroding our previous reports.[3]

Drug loading content (DLC) and entrapment efficiencies (EE) of $TCNP_{Pt}$.

To determine drug loading content (DLC) and entrapment efficiencies (EE), the prepared $TCNP_{Pt}$ solution with $mPEG_{45}$-*TK*-Ce6/PLA_{40}/Pt(IV) feed ratio of 5.0:5.0:2.5 (wt./wt.) was lyophilized. The lyophilized nanoparticles were weighted for the mass of $TCNP_{Pt}$. Then, obtained $TCNP_{Pt}$ was dissloved in DMSO. and then its the Ce6 content was determined on ultraviolet spectrophotometer (HITACHI U-5100, Tokyo, Japan) by measuring absorbance at 405 nm according to a standard curve of free Ce6 in the same DMSO solution. The platinum content of obtained $TCNP_{Pt}$ was measured on an X Series 2 Inductively Coupled Plasma Mass Spectrometer (ICP-MS, Thermo Fisher Scientific, USA). The DLC and EE were calculated from the following equations:

DLC (%) = (weight of drug in nanoparticle/weight of drug loaded nanoparticle) × 100%

EE (%) = (weight of drug in nanoparticle/weight of drug in feeding) × 100%

According to the method mentioned above, the DLCs of Ce6 and platinum were 7.5 ± 0.59% and 4.31 ± 0.36%, and the corresponding EEs were 71.2% and 56.8%.

The generation of ROS *in vitro*.

$TCNP_{Pt}$ and free Ce6 ([Ce6] = 10 μg/mL, 2 mL of each solution) were mixed with 50 mM p-nitroso-dimethylaniline (RNO) and 100 mM imidazole in 20 mM phosphate buffer (pH 7.4), and then irradiated by 660 nm laser at the light power density of 0.05

W/cm^2 for different periods of time (0, 5, 10 and 20 min). The bleaching of RNO absorption at 440 nm was determined to reflect the production of ROS.

***In vitro* drug release under 660 nm laser irradiation.**

To study the cisplatin release after continuous 660 nm laser irradiation, $TCNP_{Pt}$ solution (2 mL, [platinum] = 43.1 μg/mL, [Ce6] = 75 μg/mL) was suspended in phosphate buffer (PB buffer, 0.01 M, pH 7.4) and exposed to 660 nm laser at power density of 0.05 W/cm^2 for different time (0, 5, 10 and 20 min), and then the samples were transferred into the dialysis bag (MWCO=3500 Da) and suspended in 15 mL PB buffer (0.01 M, pH=7.4) at 37 °C with mild shaking. External PB buffer was collected at different periods, the external PB buffer was replaced with equal volume of fresh PB buffer. The collected solution was freeze-dried and analyzed by inductively coupled plasma mass spectrometer (ICP-MS) (PerkinElmer, Waltham, MA, USA).

Cell culture and animals.

The human breast cancer cell line MDA-MB-231 obtained from the American Type Culture Collection (ATCC) were cultured in complete DMEM medium containing 10% (v/v) fetal bovine serum (FBS, Gibco BRL, Eggenstein, Germany) at 37 ℃ with 5% CO_2 atmosphere. BALB/c nude mice (female, 6-weeks-old) and ICR mice (female, 6-weeks-old) were purchased from Vital River Laboratory Animal Technology Co., Ltd. (Beijing, China). All animals received care in compliance with the guidelines outlined in the Guide for the Care and Use of Laboratory Animals.

The orthotopic models of human breast cancer was established by injecting 2.0 × 10^6 MDA-MB-231 cells (100 μL) with 10% Matrigel® Matrix (Corning, Bedford, MA)

into the second mammary fat pad of mice, when tumor volumes reach about 100 mm^3, mice were used for subsequent experiments.

Pharmacokinetic studies.

Female ICR mice (6-weeks-old) were intravenously (*i.v.*) injected with free cisplatin and $TCNP_{Pt}$ (2.0 mg platinum per kg mouse weight, n = 3). At the predetermined timepoints, blood samples were collected for ICP-MS analysis.

Statistical Analysis. The statistical significance of treatment outcomes was assessed using a Student's *t*-test; $*p < 0.05$ and $**p < 0.01$ were considered statistically significant in all analyses.

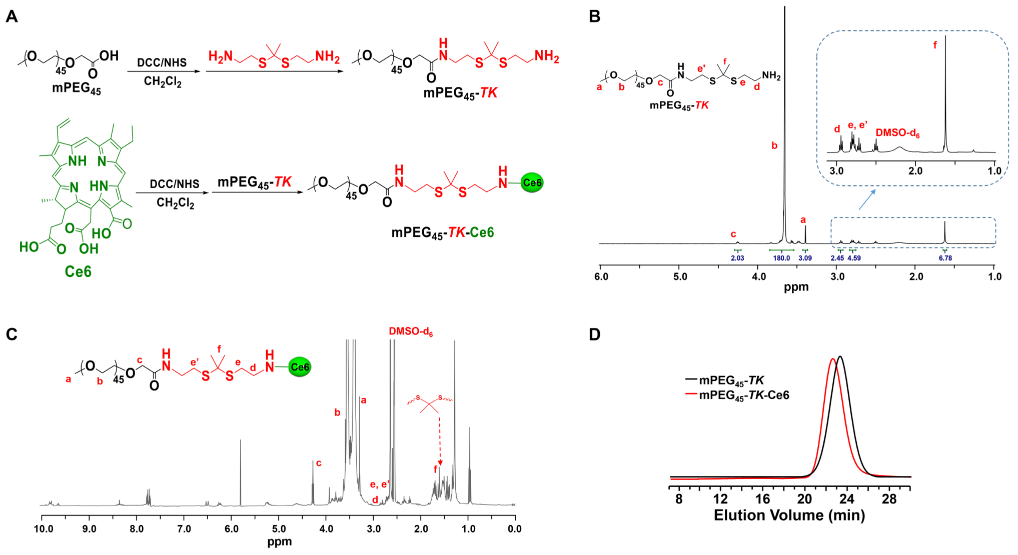

Figure S1. (A) Synthesis route of $mPEG_{45}$-*TK*-Ce6 polymer. (B) ^{1}H NMR of $mPEG_{45}$-*TK* in $CDCl_3$ recorded on an NMR Bruker AVANCE III 400 MHz spectrometer. (C) ^{1}H NMR of $mPEG_{45}$-*TK*-Ce6 in DMSO-d_6 recorded on an NMR

Bruker AVANCE III 400 MHz spectrometer. (D) GPC spectra of $mPEG_{45}$-*TK* and $mPEG_{45}$-*TK*-Ce6.

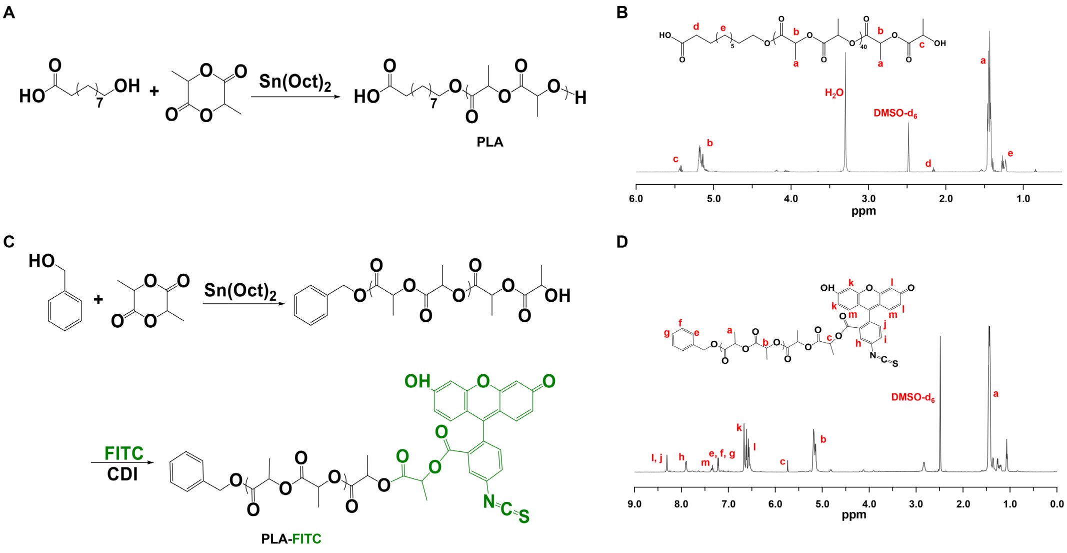

Figure S2. (A) Synthesis route of PLA. (B) ^{1}H NMR of PLA homopolymer in DMSO-d_6 recorded on an NMR Bruker AVANCE III 400 MHz spectrometer. (C) Synthesis route of PLA-FITC. (D) ^{1}H NMR of PLA-FITC in DMSO-d_6 recorded on an NMR Bruker AVANCE III 400 MHz spectrometer.

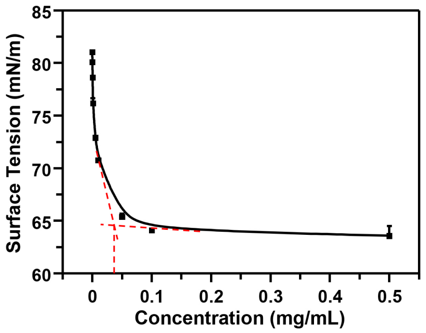

Figure S3. The surface tension of mPEG-*TK*-Ce6 in aqueous solution at various concentrations.

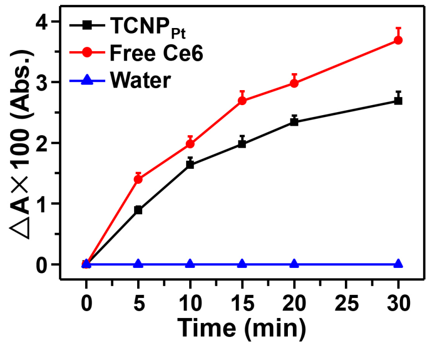

Figure S4. The generation of ROS was determined by bleaching of RNO abosorbance at 440 nm *in vitro* after 660-nm different irradiation times (0, 5, 10, 15, 20, 30 min, 0.05 W/cm^2).

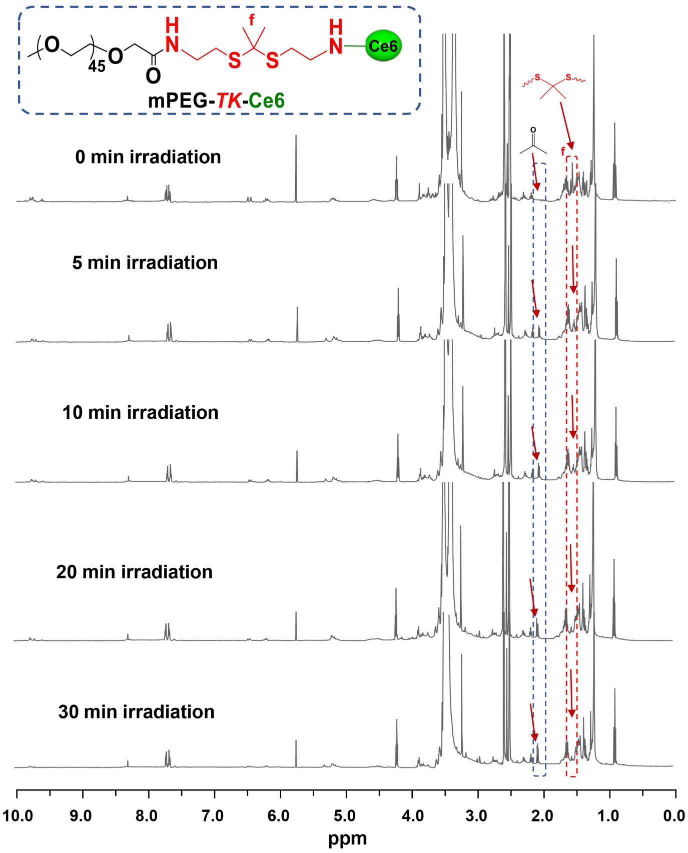

Figure S5. ^{1}H NMR of mPEG$_{45}$-*TK*-Ce6 after 660-nm laser irradiation for different times at the power intensity of 0.05 W/cm^2.

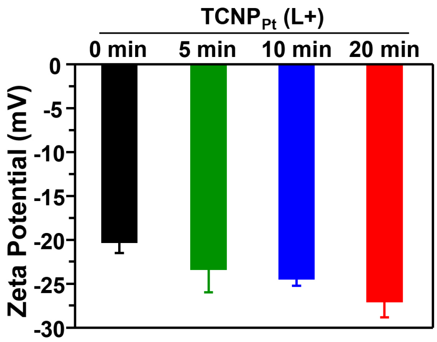

Figure S6. The zeta potential of $TCNP_{Pt}$ after irradiation for different amounts of time.

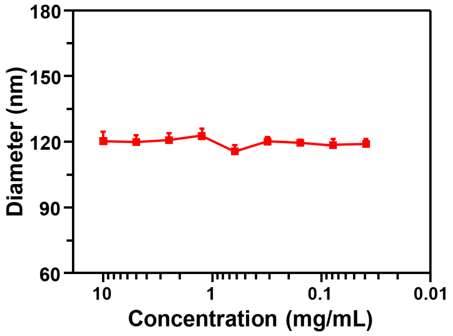

Figure S7. The dilution stability of $TCNP_{Pt}$ at various dilution concentrations in PBS.

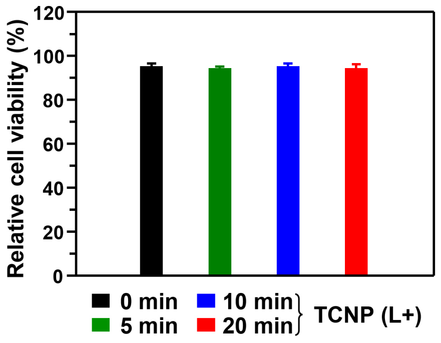

Figure S8. Cytotoxicity of blank nanoparticle TCNP (0.1 mg/mL) in MDA-MB-231 cells with preirradiation for different periods.

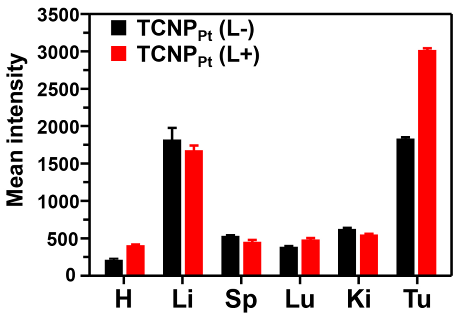

Figure S9. Mean fluorescence intensity in the tumor site according to fluorescence images in Figure 5C.

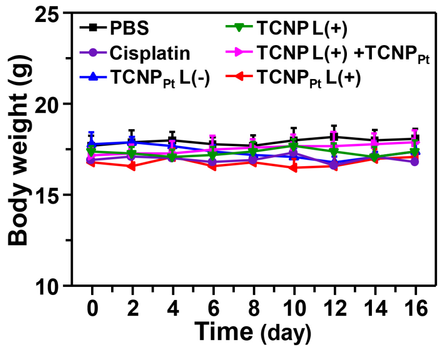

Figure S10. The body weight of MDA-MB-231 xenograft tumor-bearing mice monitored during the whole treatment. Data were presented as mean ± SD, n=5.

Table S1. Pharmacokinetic parameters of these formulations after intravenous administration (n = 3 per group).

Parameter	C_{max} (μg/mL)	T_{max} (h)	Cl (mL/h*kg)	$AUC_{0\text{-}48h}$ (μg/mL*h)
$TCNP_{Pt}$	69.9 ± 2.1	0.083	5.3 ± 0.5	354.4 ± 40.5
Cisplatin	11.9 ± 0.6	0.083	39.2 ± 2.1	33.9 ± 4.7

C_{max}, Peak concentration;

T_{max}, Time at maximum concentration;

Cl, Clearance rate;

AUC, Area under curve;

References:

1. Xu X, Xie K, Zhanga XQ, Pridgen EM, Park GY, Cui DS, et al. Enhancing tumor cell response to chemotherapy through nanoparticle-mediated codelivery of siRNA and cisplatin prodrug. Proc Natl Acad Sci U S A. 2013; 110: 18638-18643.

2. Sun CY, Cao ZY, Zhang XJ, Sun R, Yu CS, Yang XZ. Cascade-amplifying synergistic effects of chemo-photodynamic therapy using ROS-responsive polymeric nanocarriers. Theranostics. 2018; 8: 2939-2953.

3. Cao ZT, Chen ZY, Sun CY, Li HJ, Wang HX, Cheng QQ, et al. Overcoming tumor resistance to cisplatin by cationic lipid-assisted prodrug nanoparticles. Biomaterials. 2016; 94: 9-19.
